# Glasgow and Its Medical Institutions

**Published:** 1892-11-05

**Authors:** 


### The Institutional Workshop.

### WITHIN THE HOSPITALS.

### GLASGOW, AND ITS MEDICAL INSTITUTIONS.

By our Special Commissioner.

Glasgow proudly claims to be the second city in the British Empire, and though its claim is disputed by Liverpool, the town is remarkable at any rate for the great progress made by its medical institutions during the last quarter of a century. It possesses at the present time the only hospital in Great Britain which, we believe, is lighted throughout by electricity, and which is ventilated, so far as its larger wards are concerned, by a remarkably simple system of artificial ventilation. This system, as far as our observation goes, is not satisfactory in the smaller wards, but seeing that the larger wards appear to be adequately ventilated, we have come to the conclusion that the relative impurity of the air in the smaller wards is due to an inadequate arrangement of the inlets and outlets, and that it cannot justly be put down to a failure of the system in force. A close examination, and several tests that we applied to the system of ventilation at the Victoria General Infirmary, convinces us that whereas the main duct is kept comparatively free from grit and dust, the smaller ducts, communicating directly with the wards, have not been protected against the intrusion of these deleterious matters. Indeed, the evidence shows that with the air enters very much that should be excluded from the wards, as the walls and ceilings eloquently testify. Having inspected the system of ventilation at the new municipal buildings, Glasgow, which seems to have suggested the plan of ventilation now in force at the Victoria Hospital, we are of opinion that the method of washing the air at the Victoria Hospital is imperfect, and might be improved. This would tend to minimise the amount of dust and blacks which at present find their way into the wards, and if the air was again washed before it entered each ward duct, and if these ducts were to be frequently cleansed, no doubt much that is now objectionable in the system would be removed. Having regard to the temperate character of the British climate, we have yet to be convinced that it is desirable or necessary to introduce artificial ventilation into our hospitals: or, indeed, that it is desirable, or even permissible, to rely entirely upon such a system for the whole ventilation of the sick wards of any British institution which is constantly filled with surgical and other cases requiring an abundance of fresh air. If these objections can be over-ruled, then, no doubt, from its simplicity and comparative cheapness, the system of ventilation in force at the Victoria General Infirmary will commend itself to the adoption of many hospital authorities in the United Kingdom. In other climates, with the modifications we have suggested, the system should prove of real service, and we commend it to the attention of those who have to construct hospitals abroad, as it is well worthy of close study.

The medical institutions at Glasgow possess some features of great interest. The additions and improvements at the Royal Infirmary, the new nursing home at the Western Infirmary, the new out-patient department at the Children's Hospital, the memorial home to the late Mrs. Higginbotham in connection with the Glasgow Sick Poor and Private Nursing Association, as well as the important departure in relation to the training of nurses, just instituted at the Royal Infirmary, all afford striking evidence of marked progress on the part of the City of Glasgow and its inhabitants. Add to all this the remarkable fact that, despite the enormous growth in the number of beds provided in recent years for the accommodation of the sick poor, owing to the rise and development of two great hospitals like the Western and the Victoria Infirmaries, and the consequent considerable increase in the expenditure, that an abundance of income from voluntary sources has been and is forthcoming, and Glasgow is undoubtedly entitled to attract the notice and excite the emulation of the inhabitants of other important cities throughout the United Kingdom.

#### The Newer Hospital Buildings.

The Victoria Infirmary, the most modern hospital in Glasgow, is built upon the pavilion plan, and contains nearly all of the best and most recent features known to hospital experts. Its wards are well-proportioned. there is abundant air and wall space, the furniture and fittings exhibit marked care and thought, the appliances are of the best kind, the lavatories and baths are of the latest pattern, and the whole establishment impresses the visitor with the feeling that those responsible for its administration have taken enormous pains, are deeply interested in the work, and have secured, as they deserve, a substantial success. So great has been the popularity of this new hospital, so liberal has been the support that it has received, and so great is the pressure upon the beds, that already new buildings have been erected, and now approach completion, which will double the accommodation. Dr. Mackintosh, the late Superintendent, and Miss Ross, the Matron, deserve great credit for the high state of efficiency which at present prevails throughout all the departments.

#### The Royal Infirmary.

This institution, from its contiguity to an old burying-ground, and the circumstance that many of the wards have been in use for a great number of years, is a remarkable institution when the results of treatment are studied. Those results reflect the greatest credit upon the medical and surgical staff, and show that the administration has seconded their efforts to the utmost. The Nurses' Home provides a separate room for each nurse, and contains one of the largest recreation rooms we have seen, though we thought it rather short of comfortable chairs and lounges. The new laundry buildings have been carefully thought out, and the machinery and labour-saving appliances in use are good. It was noteworthy that the linen, although no chemicals or other aids were used, was so beautifully white as to make a Londoner wonder how it had been possible to turn it out in a town laundry situated in the heart of a great city like Glasgow. The new kitchens, too, are excellent in all respects. Hospital managers elsewhere may usefully note that the laundry and also the kitchens have been placed under the superintendence of women who have undergone technical training, and are therefore well qualified to secure economy and to afford satisfaction to all whose interests are in any way dependent upon the efficiency of these two departments. The dining-hall for nurses and servants and the general dining-rooms are also good.

THE WESTERN INFIRMARY.

No structural alterations appear to have been made in recent years to the hospital portion of this infirmary. Our tour through the hospitals of the great manufacturing districts has impressed us with the importance of brightness and cheerfulness, secured by means of close attention to detail in connection with the decoration and the colouring of the wards and corridors. This important point seems to have escaped the attention of the managers of the Western Infirmary, and we are convinced that the new Superintendent, Dr. Mackintosh, will make a note of the point, and that the sooner the directors vote a considerable sum of money and have the whole of the corridors and wards of this institution redecorated with due regard to artistic effect, the better will it be not only for those who work and suffer within its walls, but for the finances of the institution also.

THE CHILDREN'S HOSPITAL.

The new out-patient department at the children's hospital is worthy of the closest study by all hospital authorities and experts. It presents many unique features, and exhibits an amount of thought and knowledge on the part of those who designed it highly creditable to all concerned. It consists of a central hall for out-patients with the usual physicians' and surgeons' rooms, and an inspection room and bath room, on the medical and surgical side respectively, each placed in charge of a nurse who examines and when necessary baths the patients and prepares them for examination by the medical man. Upstairs are residential chambers occupied by the distrct nurses attached to the out-patient department. These nurses follow a case home when necessary, *i.e.*, where an operation has been performed, and look after it until convalescent. Of course, Glasgow has an extensive area, and as only a limited number of nurses are employed, resort is had in prolonged cases to the Glasgow Sick Poor and Private Nursing Association who undertake the nursing of the sick poor in their own homes, and so relieve the out-patient department of this branch of its work. On the surgical side the consulting rooms contain an operation table and a bed, on which a patient can lie till recovered from the effects of an anæsthetic, should this have to be administered. On the medical side is an elaborate system of electrical appliances under the charge of a special officer, and this branch of the work seems to be done more thoroughly than at any institution with which we are acquainted. On another occasion we hope to give an illustration and full account of the electrical apparatus to be found here and at the Western Infirmary at Glasgow. The case work, and indeed the whole medical and surgical practice of this out-patient department,is full of interest and instruction. The system in force is worthy of close study, and might in many points be advantageously adopted by other children's hospitals throughout the country.

The Children's Hospital has been erected within a short walk of the out-patient department. The nursing there struck us as very good. The wards are airy and well proportioned; there is a due allowance of wall, superficial, and cubic space, and much good work is being done here. Every attention is paid to hygienic considerations. The lavatories and baths are in good order, and the whole establishment shows an immense advance upon the accommodation provided previous to the occupation of the present premises. We require more space than we have available at present to deal with the nursing institutions indicated in the course of this article, so we reserve them for future consideration.

